# Supplementary material for: Modeling glioblastoma heterogeneity as a dynamic network of cell states
Source: Mol Syst Biol. 2021 Sep 16;17(9):e10105. doi: 10.15252/msb.202010105 (PMC8444284; doi:10.15252/msb.202010105)
Supplement: Supplementary file 6 — Source Data for Figure 5 [file MSB-17-e10105-s004.zip › Figure5A_sourcedata/GSEA_3017/nosigdb_stateC.GseaPreranked.1622795002785/RICHARDS_NOWAKOWSKI_SCIENCE_2017_U1_UPREG.html]

Details for gene set RICHARDS\_NOWAKOWSKI\_SCIENCE\_2017\_U1\_UPREG[GSEA]

|  || Dataset | state13017 |
| Phenotype | NoPhenotypeAvailable |
| Upregulated in class | na\_neg |
| GeneSet | RICHARDS\_NOWAKOWSKI\_SCIENCE\_2017\_U1\_UPREG |
| Enrichment Score (ES) | -0.40502697 |
| Normalized Enrichment Score (NES) | -2.9427915 |
| Nominal p-value | 0.0 |
| FDR q-value | 0.0 |
| FWER p-Value | 0.0 |
Table: GSEA Results Summary

  

Fig 1: Enrichment plot: RICHARDS\_NOWAKOWSKI\_SCIENCE\_2017\_U1\_UPREG      
 Profile of the Running ES Score & Positions of GeneSet Members on the Rank Ordered List

  

| PROBE | GENE SYMBOL | GENE\_TITLE | RANK IN GENE LIST | RANK METRIC SCORE | RUNNING ES | CORE ENRICHMENT || 1 | CDK6 |  |  | 24 | 0.518 | -0.0414 | No |
| 2 | GAS1 |  |  | 64 | 0.384 | -0.1178 | No |
| 3 | TCF12 |  |  | 71 | 0.376 | -0.1233 | No |
| 4 | CSRP2 |  |  | 72 | 0.376 | -0.1158 | No |
| 5 | NASP |  |  | 75 | 0.371 | -0.1127 | No |
| 6 | MPP6 |  |  | 82 | 0.360 | -0.1184 | No |
| 7 | RHOBTB3 |  |  | 124 | 0.313 | -0.2006 | No |
| 8 | SNCAIP |  |  | 182 | 0.264 | -0.3182 | No |
| 9 | GALNT10 |  |  | 197 | -0.251 | -0.3433 | No |
| 10 | CAST |  |  | 205 | -0.253 | -0.3534 | No |
| 11 | FERMT2 |  |  | 227 | -0.259 | -0.3935 | No |
| 12 | ATOX1 |  |  | 229 | -0.260 | -0.3905 | No |
| 13 | MBNL2 |  |  | 233 | -0.260 | -0.3918 | No |
| 14 | CLIC1 |  |  | 237 | -0.261 | -0.3930 | No |
| 15 | CCDC6 |  |  | 240 | -0.262 | -0.3921 | No |
| 16 | SAT1 |  |  | 241 | -0.262 | -0.3869 | No |
| 17 | ZFYVE21 |  |  | 247 | -0.263 | -0.3924 | No |
| 18 | TPT1 |  |  | 248 | -0.263 | -0.3872 | No |
| 19 | MYOF |  |  | 251 | -0.263 | -0.3863 | No |
| 20 | NPAS3 |  |  | 256 | -0.265 | -0.3896 | No |
| 21 | RUNX2 |  |  | 260 | -0.267 | -0.3908 | No |
| 22 | PPIB |  |  | 265 | -0.268 | -0.3940 | No |
| 23 | AMOT |  |  | 267 | -0.268 | -0.3909 | No |
| 24 | ANAPC16 |  |  | 268 | -0.269 | -0.3855 | No |
| 25 | RPL41 |  |  | 270 | -0.269 | -0.3823 | No |
| 26 | MEG3 |  |  | 273 | -0.270 | -0.3812 | No |
| 27 | SOCS3 |  |  | 274 | -0.270 | -0.3759 | No |
| 28 | EHD2 |  |  | 275 | -0.270 | -0.3705 | No |
| 29 | SULF2 |  |  | 276 | -0.270 | -0.3651 | No |
| 30 | HEATR5A |  |  | 278 | -0.271 | -0.3619 | No |
| 31 | RRBP1 |  |  | 283 | -0.271 | -0.3651 | No |
| 32 | UGCG |  |  | 287 | -0.273 | -0.3661 | No |
| 33 | RPL23 |  |  | 288 | -0.273 | -0.3607 | No |
| 34 | PDGFA |  |  | 297 | -0.275 | -0.3725 | No |
| 35 | RPS18 |  |  | 301 | -0.277 | -0.3734 | No |
| 36 | HERPUD1 |  |  | 302 | -0.277 | -0.3679 | No |
| 37 | PAPPA |  |  | 304 | -0.279 | -0.3645 | No |
| 38 | NAMPT |  |  | 306 | -0.280 | -0.3611 | No |
| 39 | PDLIM2 |  |  | 314 | -0.283 | -0.3705 | No |
| 40 | CRISPLD1 |  |  | 331 | -0.288 | -0.3993 | Yes |
| 41 | RPL12 |  |  | 332 | -0.288 | -0.3936 | Yes |
| 42 | GNB4 |  |  | 335 | -0.289 | -0.3921 | Yes |
| 43 | NRBF2 |  |  | 337 | -0.289 | -0.3885 | Yes |
| 44 | ARMCX2 |  |  | 338 | -0.290 | -0.3827 | Yes |
| 45 | SPATS2L |  |  | 339 | -0.290 | -0.3770 | Yes |
| 46 | MMP2 |  |  | 340 | -0.291 | -0.3712 | Yes |
| 47 | PPP1R15A |  |  | 342 | -0.293 | -0.3675 | Yes |
| 48 | RPS2 |  |  | 346 | -0.296 | -0.3681 | Yes |
| 49 | DLC1 |  |  | 347 | -0.296 | -0.3622 | Yes |
| 50 | MEST |  |  | 352 | -0.299 | -0.3648 | Yes |
| 51 | ITGB5 |  |  | 356 | -0.300 | -0.3653 | Yes |
| 52 | CMBL |  |  | 358 | -0.301 | -0.3615 | Yes |
| 53 | UGDH |  |  | 360 | -0.301 | -0.3576 | Yes |
| 54 | S100A11 |  |  | 361 | -0.301 | -0.3516 | Yes |
| 55 | RPL28 |  |  | 363 | -0.302 | -0.3478 | Yes |
| 56 | LRP1 |  |  | 366 | -0.304 | -0.3460 | Yes |
| 57 | PMEPA1 |  |  | 375 | -0.308 | -0.3571 | Yes |
| 58 | LMNA |  |  | 376 | -0.308 | -0.3510 | Yes |
| 59 | FOS |  |  | 377 | -0.309 | -0.3449 | Yes |
| 60 | APLP2 |  |  | 379 | -0.310 | -0.3409 | Yes |
| 61 | IGFBP5 |  |  | 384 | -0.311 | -0.3433 | Yes |
| 62 | SAMD4A |  |  | 388 | -0.313 | -0.3435 | Yes |
| 63 | CETN2 |  |  | 392 | -0.317 | -0.3437 | Yes |
| 64 | LRRC4C |  |  | 394 | -0.318 | -0.3395 | Yes |
| 65 | S100A4 |  |  | 395 | -0.318 | -0.3332 | Yes |
| 66 | S100A13 |  |  | 396 | -0.319 | -0.3268 | Yes |
| 67 | TIMP1 |  |  | 399 | -0.320 | -0.3247 | Yes |
| 68 | ZFP36L2 |  |  | 401 | -0.321 | -0.3205 | Yes |
| 69 | SEMA3A |  |  | 406 | -0.325 | -0.3227 | Yes |
| 70 | TPM1 |  |  | 407 | -0.327 | -0.3162 | Yes |
| 71 | GAS5 |  |  | 411 | -0.329 | -0.3161 | Yes |
| 72 | SIPA1L1 |  |  | 412 | -0.329 | -0.3095 | Yes |
| 73 | DDX21 |  |  | 413 | -0.331 | -0.3029 | Yes |
| 74 | LDLR |  |  | 419 | -0.334 | -0.3071 | Yes |
| 75 | CALD1 |  |  | 420 | -0.335 | -0.3004 | Yes |
| 76 | MYC |  |  | 421 | -0.336 | -0.2937 | Yes |
| 77 | SLC2A3 |  |  | 422 | -0.336 | -0.2870 | Yes |
| 78 | S100A6 |  |  | 423 | -0.336 | -0.2803 | Yes |
| 79 | RHOQ |  |  | 426 | -0.338 | -0.2779 | Yes |
| 80 | KLF6 |  |  | 429 | -0.339 | -0.2755 | Yes |
| 81 | SNHG16 |  |  | 431 | -0.340 | -0.2708 | Yes |
| 82 | REXO2 |  |  | 432 | -0.342 | -0.2640 | Yes |
| 83 | WWTR1 |  |  | 437 | -0.345 | -0.2658 | Yes |
| 84 | WLS |  |  | 438 | -0.345 | -0.2589 | Yes |
| 85 | PCDH9 |  |  | 439 | -0.345 | -0.2520 | Yes |
| 86 | FAT1 |  |  | 441 | -0.346 | -0.2473 | Yes |
| 87 | RAB31 |  |  | 445 | -0.349 | -0.2468 | Yes |
| 88 | HSPA5 |  |  | 447 | -0.350 | -0.2420 | Yes |
| 89 | MYL12A |  |  | 448 | -0.350 | -0.2350 | Yes |
| 90 | SERPINE2 |  |  | 452 | -0.355 | -0.2344 | Yes |
| 91 | DKK3 |  |  | 454 | -0.356 | -0.2295 | Yes |
| 92 | RHOC |  |  | 455 | -0.359 | -0.2224 | Yes |
| 93 | FLRT3 |  |  | 461 | -0.363 | -0.2259 | Yes |
| 94 | SERPINB6 |  |  | 463 | -0.367 | -0.2208 | Yes |
| 95 | ALCAM |  |  | 467 | -0.370 | -0.2198 | Yes |
| 96 | B2M |  |  | 468 | -0.371 | -0.2125 | Yes |
| 97 | LGALS3 |  |  | 471 | -0.374 | -0.2093 | Yes |
| 98 | COL9A3 |  |  | 477 | -0.379 | -0.2126 | Yes |
| 99 | FIBIN |  |  | 478 | -0.379 | -0.2050 | Yes |
| 100 | RAB13 |  |  | 482 | -0.388 | -0.2038 | Yes |
| 101 | IFITM2 |  |  | 483 | -0.388 | -0.1960 | Yes |
| 102 | VCL |  |  | 484 | -0.388 | -0.1883 | Yes |
| 103 | ARL6IP5 |  |  | 490 | -0.396 | -0.1912 | Yes |
| 104 | PLIN3 |  |  | 496 | -0.411 | -0.1938 | Yes |
| 105 | LDHA |  |  | 498 | -0.412 | -0.1877 | Yes |
| 106 | JUN |  |  | 503 | -0.425 | -0.1879 | Yes |
| 107 | PRSS23 |  |  | 510 | -0.438 | -0.1921 | Yes |
| 108 | BCAT1 |  |  | 511 | -0.438 | -0.1834 | Yes |
| 109 | PTN |  |  | 517 | -0.446 | -0.1853 | Yes |
| 110 | AHNAK |  |  | 519 | -0.449 | -0.1785 | Yes |
| 111 | CEBPD |  |  | 523 | -0.455 | -0.1759 | Yes |
| 112 | IFITM3 |  |  | 533 | -0.477 | -0.1858 | Yes |
| 113 | COL6A1 |  |  | 534 | -0.479 | -0.1763 | Yes |
| 114 | LRRN3 |  |  | 543 | -0.504 | -0.1835 | Yes |
| 115 | SNHG3 |  |  | 549 | -0.518 | -0.1840 | Yes |
| 116 | GJA1 |  |  | 552 | -0.527 | -0.1778 | Yes |
| 117 | ANXA2 |  |  | 563 | -0.567 | -0.1880 | Yes |
| 118 | TNC |  |  | 564 | -0.572 | -0.1766 | Yes |
| 119 | SPARC |  |  | 567 | -0.575 | -0.1695 | Yes |
| 120 | EMP3 |  |  | 568 | -0.580 | -0.1579 | Yes |
| 121 | S100A16 |  |  | 569 | -0.582 | -0.1464 | Yes |
| 122 | LAMA4 |  |  | 571 | -0.620 | -0.1362 | Yes |
| 123 | CD44 |  |  | 575 | -0.659 | -0.1295 | Yes |
| 124 | VIM |  |  | 578 | -0.674 | -0.1204 | Yes |
| 125 | CLU |  |  | 581 | -0.690 | -0.1110 | Yes |
| 126 | S100A10 |  |  | 582 | -0.691 | -0.0972 | Yes |
| 127 | CYR61 |  |  | 584 | -0.714 | -0.0852 | Yes |
| 128 | CA12 |  |  | 586 | -0.717 | -0.0730 | Yes |
| 129 | HAS2 |  |  | 587 | -0.740 | -0.0583 | Yes |
| 130 | CTGF |  |  | 588 | -0.782 | -0.0427 | Yes |
| 131 | EMP1 |  |  | 589 | -0.785 | -0.0271 | Yes |
| 132 | PMP22 |  |  | 590 | -0.837 | -0.0105 | Yes |
| 133 | CCL2 |  |  | 595 | -1.066 | 0.0022 | Yes |
Table: GSEA details [plain text format]

  

Fig 2: RICHARDS\_NOWAKOWSKI\_SCIENCE\_2017\_U1\_UPREG: Random ES distribution      
 Gene set null distribution of ES for **RICHARDS\_NOWAKOWSKI\_SCIENCE\_2017\_U1\_UPREG**
